# Triglyceride-to-glucose index to detect a non-dipping circadian pattern in newly diagnosed hypertensive patients

**DOI:** 10.34172/jcvtr.2022.20

**Published:** 2022-06-25

**Authors:** Faysal Şaylık, Tufan Çınar, Murat Selçuk, Tayyar Akbulut

**Affiliations:** ^1^Department of Cardiology, Van Training and Research Hospital, 65100, Van, Turkey; ^2^Department of Cardiology, Sultan 2. Abdulhamid Han Training and Research Hospital, 34100, Istanbul, Turkey

**Keywords:** Triglyceride-Glucose Index, Non-Dipping Hypertension, Insulin Resistance, Biomarker, Circadian Pattern

## Abstract

***Introduction: *** In this investigation, we aimed to explore the relationship between the triglyceride-glucose (TyG) index and the non-dipping blood pressure (BP) pattern in newly diagnosed hypertensive patients.

***Methods:*** In this retrospective study, 216 consecutive newly diagnosed hypertensive patients who had undergone 24-hour ambulatory blood pressure (ABPM) monitoring and had not received anti-hypertensive drugs were included. Non-dipping status was evaluated by a 24-h ABPM monitoring in all patients. We categorized the patients into two groups as; dippers (n=104 cases) and non-dippers (n=112 cases). The TyG index was derived from the fasting triglyceride and fasting glucose levels using the formula; ln[fasting triglyceride (mg/dL) × fasting glucose (mg/dL)/2].

***Results:*** Non-dipper group had a higher TyG index than the dipper group. The TyG was an independent predictor of non-dipping BP in hypertensive individuals, according to multivariable analysis. The TyG index was negatively associated with a decrease in both systolic and diastolic BP during the nighttime. The ideal cutoff value of the TyG index in detecting non-dipping status was≥9.01 with 74.1% sensitivity and 71.2% specificity. A ROC comparison indicated that the area under the curve value of TyG index was superior to fasting triglyceride, fasting glucose, and homeostasis model assessment of IR (HOMA-IR) in detecting non-dipping BP.

***Conclusion:*** The TyG index was an independent predictor of non-dipping status in newly diagnosed hypertensive patients who had undergone 24-hour ABPM monitoring and had not received anti-hypertensive drugs. As a simple and easily obtained parameter, the TyG index can be used to detect such pattern among these patients.

## Introduction

 Dipper-pattern (DP) blood pressure (BP) is described as a both systolic and diastolic BP fall of more than 10% during the nighttime compared to daytime.^1^ On the other hand, a fail to decrease more than 10% in BP during the nighttime is referred to as non-dipper pattern (non-DP) BP, which is linked to adverse cardiovascular events and target organ damage.^2^ Thus, 24-hour ambulatory blood pressure monitoring (ABPM) is often performed to detect the lack of this variability in high-risk patients.

 Insulin resistance (IR) is a pathological status in which insulin has a lower biological effect than expected. IR is thought to be a risk factor for heart disease.^3^ Moreover, the association of IR with hypertension had been established in previous studies, and a higher prevalence of non-DP was found in patients with IR.^4^ Triglyceride-glucose (TyG) index has emerged as a useful marker of IR that is calculated based on fasting glucose and triglyceride.^5^ The TyG index is found to be superior to the homeostasis model assessment of IR (HOMA-IR) for assessing IR.^6^ The association of the TyG index with cardiovascular diseases, including coronary artery disease, hypertension, arterial stiffness, and carotid atherosclerosis, was reported in previous studies.^7-9^ However, no prior study has evaluated the association of the TyG index with the circadian pattern of BP. Thus, the goal of this study was to assess if there was a link between the TyG index and non-DP in newly diagnosed hypertension patients who were not on antihypertensive treatment.

## Materials and Methods

###  Data collection

 In all, 216 newly-diagnosed treatment-naive hypertensive patients who had both clinical and 24-hour ABPM assessments at the cardiology outpatient clinic between January 2015 and March 2020 were included in this retrospective, cross-sectional study. Hypertension was defined as two or more measures of systolic blood pressure (≥140 mmHg) and/or diastolic blood pressure (≥90 mmHg). on separate days and the mean 24-hour ABPM SBP ≥ 130 mmHg and/or the mean 24-h ABPM DBP ≥ 80 mmHg or the mean daytime 24-hour ABPM SBP ≥ 135 mmHg and/or the mean daytime 24-hour ABPM DBP ≥ 85 mmHg as recommended in a recent guideline published by European Society of Cardiology.^1^ Patients with a high clinical BP who underwent a 24-hour ABPM were included in the study. The exclusion criteria were as the followings; patients who were diagnosed with hypertension previously and/or used anti-hypertensive treatment and those who were treated with anti-hyperlipidemic or anti-hyperglycemic drugs, had diabetes mellitus, coronary artery disease, heart failure, creatinine level above 1.5 mg/dL, hepatic disease, acute or chronic infectious disease, inflammatory disease, and malignancy. The current study was carried out in accordance with the Declaration of Helsinki, version 2008.

###  Laboratory analysis

 After a 12-hour overnight fast, all blood samples were taken in the morning. The Coulter LH 750 analyzer (Beckman Coulter, Galway, Ireland) was used to assess the total blood count parameters. The following formula was used to determine the TyG index; TyG index = ln (fasting triglyceride (mg/dL) x fasting glucose (mg/dL)/2).^7^ HOMA-IR was calculated as; HOMA-IR = fasting insulin (microU/L) x fasting glucose (mg/dL)/405.^10^

###  Blood pressure measurement 

 The BP measurement of each patient was performed two or more times on separate days after at least 10 minutes of rest at the cardiology outpatient clinic. Patients with high clinical BP (mean SBP ≥140 mmHg and/or mean DBP ≥90 mmHg) underwent 24-hour ABPM.

###  24-h ABPM

 A 24-hour ABPM (Schiller MT-300 BP, Baar, Switzerland), which recorded BP and pulse rate in the non-dominant arm at 15-minute intervals in the daytime and at 30-minute intervals at nighttime, was performed in patients with high BP. The daytime was referred to the time interval between 06:00 A.M. and 10:00 P.M., and the nighttime was referred to the time interval between 10:00 P.M. and 06:00 A.M. In patients whose acceptable measurements in daytime and nighttime were below 70%, second 24-hour ABPM was conducted. DP was accepted as a 10% or more decrease in BP during the nighttime period compared to daytime, whereas non-DP was accepted as less than a 10% decrease in BP in that period.^11^

###  Statistical Analysis 

 All analyses were performed using R-Studio Version 4.0.3 (RStudio, Boston, MA, USA).

 The normality of the data was determined using the Kolmogorov-Smirnov test. Categorical variables were presented as numbers and percentages. Quantitative variables with a normal distribution were reported as mean (standard deviation) and those without normal distribution as median (25-75^th^ interquartile range). The statistical differences in continuous variables between the groups were calculated using an independent Student’s t-test or a Mann-Whitney U test. To compare categorical variables, the chi-square test or Fisher’s exact test were used, as applicable. The independent determinants of non-DP status were determined using univariate and multivariate logistic regression analysis. The model in the multivariable logistic regression analysis was created with the variables that were statistically significant in the univariable logistic regression analysis. To avoid multicollinearity and interaction, fasting glucose, fasting triglyceride, and fasting insulin were not included in the multivariate model with TyG index and HOMA-IR. There were no additional variables with multicollinearity in the model. Receiver operating characteristic (ROC) curve analysis was employed to detect the optimal cutoff value for the TyG index in detecting patients with non-DP status using the Youden index. ROC curve comparisons were computed using the DeLong test between TyG index, triglyceride, glucose, and HOMA-IR to compare the discrimination ability of those variables for non-DP in hypertensive patients. Spearman rank-correlation analyses were performed to determine the associations between the TyG index and the declines of both SBP and DBP from daytime to nighttime. We calculated a-priori required minimum total sample size as 98 with an effect size of 0.57 with 80 % power and 0.05 alfa error probability by calculating the effect size based on a previous report.^12^ Thus, we conducted this study with 216 patients. A post-hoc study power was calculated as 99% with 0.98 effect size for our study. Statistical significance was defined as a two-sided *P* value < 0.05.

## Results

 The study population compromised 216 patients who were categorized into two groups according to 24-hour ABPM as DP (n = 104 cases, 62.5% male) and with non-DP (n = 112 cases, 56.2% male). The non-DP group had higher fasting glucose, triglyceride, TyG index, clinical SBP and DBP, low-density lipoprotein (LDL) cholesterol, fasting insulin, HOMA-IR, and red cell distribution width (RDW) compared to the DP group. The other characteristics of patients were given in Table 1.

**Table 1:** Basal characteristics of patients with dipper and non-dipper hypertension

	**Dipper pattern (n=104)**	**Non-dipper pattern (n=112)**	***P*** ** value**
Age, years	51.1 (12.0)	50.7 (12.4)	0.829^*^
Men, n (%)	65 (62.5)	63 (56.2)	0.426^&^
Current smoker, n (%)	27 (26.0)	32 (28.6)	0.782^&^
BMI, kg/m^2^	27.4 (3.57)	28.0 (4.10)	0.219^*^
Clinical SBP, mmHg	143 (8.88)	146 (11.4)	**0.034** ^*^
Clinical DBP, mmHg	90.9 (6.29)	92.9 (7.64)	**0.031** ^*^
Fasting glucose, mg/dL	92.5 (2.5)	94.2(3.6)	**<0.001** ^*^
Fasting insulin	10.9(9.1-12.3)	11.5(10.1-14.6)	**0.003** ^#^
HOMA-IR	2.2(1.4-2.8)	2.5(1.9-3.2)	**<0.001** ^#^
Creatinine, mg/dL	0.75(0.18)	0.74(0.13)	0.719^*^
**Fasting lipid status, mg/dL**			
Total cholesterol	189 (28.3)	187 (32.3)	0.602^*^
HDL-cholesterol	45.0 (40.0-53.9)	46.0 (37.8-56.0)	0.844^#^
LDL-cholesterol	104(30)	117(29)	**0.002** ^*^
Triglycerides	148 (101-184)	174 (151-218)	**<0.001** ^#^
TyG index	8.82(8.5-9.08)	9.15(8.96-9.42)	**<0.001** ^#^
WBC, x 10^9^/L	7.72 (1.83)	7.46 (1.65)	0.272^*^
Hemoglobin, g/L	14.5(1.3)	14.9(1.8)	0.07^*^
Platelet, x 10^9^/L	275 (60.2)	270 (56.4)	0.512^*^
MCV, fL	87.1(5.4)	88.6(6.4)	0.06^*^
RDW, %	13.2(12.6-13.6)	13.5(13.1-13.9)	**<0.001** ^#^

Abbreviations: BMI, body mass index; DBP, diastolic blood pressure, HDL, high-density cholesterol; HOMA-IR, homeostasis model assessment of insulin resistance; LDL, low-density cholesterol; MCV, mean corpuscular volume; RDW, red cell distribution width; SBP, systolic blood pressure; TyG, triglyceride-glucose index; WBC, white blood cell. Continuous variables with normal distribution are presented as mean (standard deviation) and those with non-normal distribution as median (interquartile range). Categorical variables are presented as numbers (%). * Independent sample t-test was used for comparison between groups. # Mann-Whitney U test was used for comparison between groups. & Chi-squared test was used for comparison between groups.

**Figure 1:**
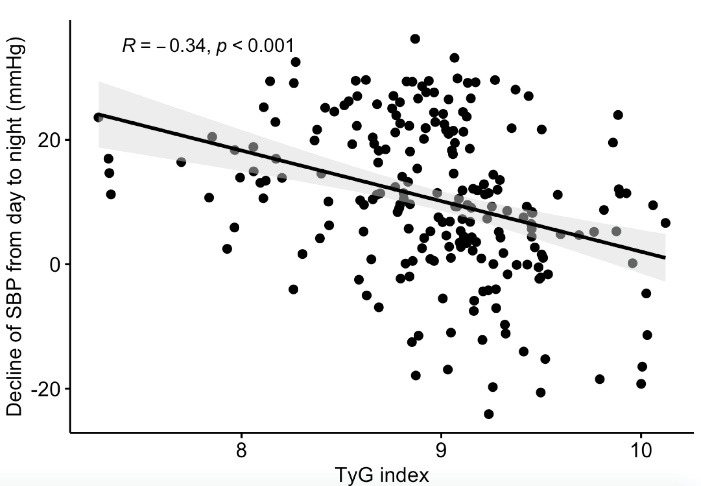
The correlation plot of the TyG index with the decline of SBP from day to night. Abbreviations; SBP: systolic blood pressure, TyG: triglyceride-glucose.

**Figure 2:**
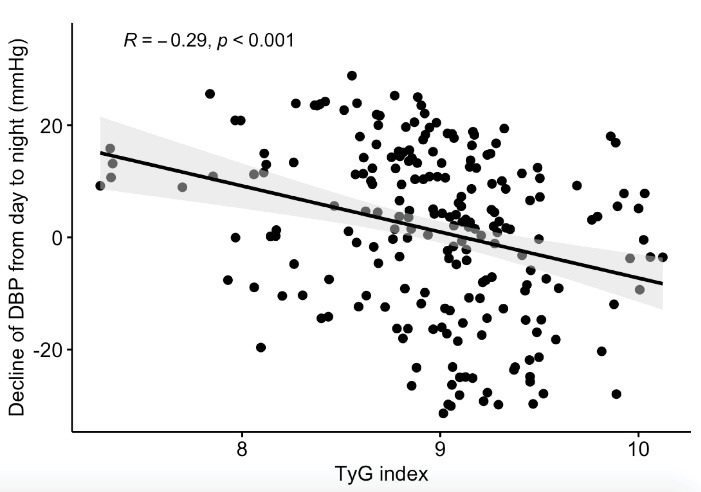
The correlation plot of the TyG index with the decline of DBP from day to night. Abbreviations; DBP: diastolic blood pressure, TyG: triglyceride-glucose.

**Figure 3:**
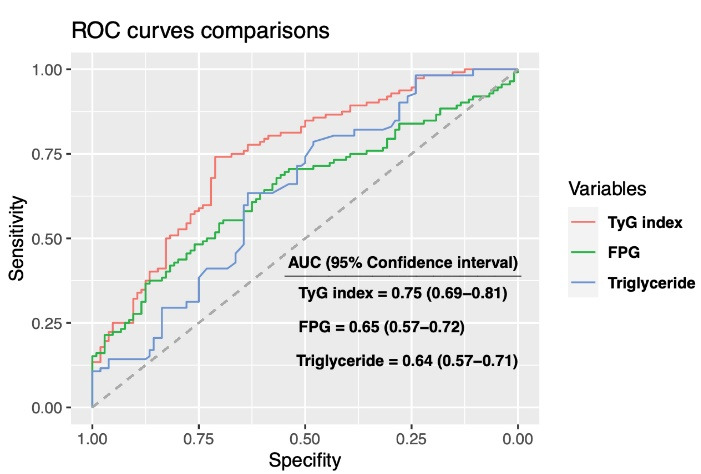
ROC curves comparisons of fasting triglyceride, fasting glucose, and the TyG index. Abbreviations; ROC: receiver operating curve, TyG: triglyceride-glucose.

**Figure 4:**
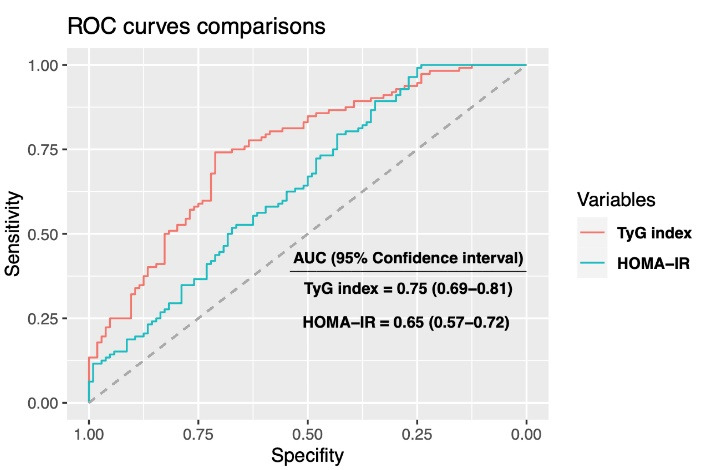
ROC curves comparisons of HOMA-IR and the TyG index. Abbreviations; HOMA-IR: homeostasis model assessment of insulin resistance, ROC: receiver operating curve, TyG: triglyceride-glucose.

 24-hour ABPM results were demonstrated in Table 2. The non-DP group had higher values of 24-hour mean BP, nighttime SBP, nighttime DBP, and nighttime mean BP than the DP group. Clinical SBP, TyG index, HOMA-IR, RDW, LDL cholesterol, and 24-hour mean BP were independent predictors of non-DP status in hypertensive patients (Table 3). Spearman correlation analysis was remarkable with negatively significant correlation of TyG index with a decrease in both SBP (R = -0.34, *P* < 0.001, Figure 1) and DBP (R = -0.29, *P* < 0.001, Figure 2). The results of the ROC analysis revealed that the ideal cutoff point of the TyG index in determining non-dipping status was ≥ 9.01 with 74.1% sensitivity and 71.2 % specificity. In ROC comparisons, the area under curve (AUC) value of TyG index was superior to fasting triglyceride, fasting glucose (Figure 3), and HOMA-IR (Figure 4) for detecting non-DP in hypertensive patients. The Delong test comparison revealed statistically significant differences between TyG index and HOMA-IR (*P* = 0.035), triglyceride (*P* = 0.029), and glucose (*P* = 0.036).

**Table 2:** 24-hour ambulatory blood pressure monitoring values of dipper and non-dipper groups.

	**Dipper pattern (n=104)**	**Non-dipper pattern (n=112)**	***P*** ^*^ **value**
24-hour SBP (mmHg)	133.7(14.6)	134.9(16)	0.561
24-hour DBP (mmHg)	83.9(11.2)	86(13.3)	0.213
24-hour mean BP (mmHg)	101.2(7.1)	107.3(7.8)	**<0.001**
Daytime SBP (mmHg)	133.2(13.5)	136.7(16.2)	0.083
Daytime DBP (mmHg)	87.1(11.4)	88(14.3)	0.598
Daytime mean BP (mmHg)	106.9(7.6)	107.8(8.8)	0.439
Nighttime SBP (mmHg)	116.4(13.4)	132.1(16.7)	**<0.001**
Nighttime DBP (mmHg)	75.1(9.4)	83.5(11.9)	**<0.001**
Nighttime mean BP (mmHg)	88.9(7.8)	99.9(8.5)	**<0.001**

Abbreviations: DBP, diastolic blood pressure; SBP, systolic blood pressure. * Independent sample t-test was used for comparison between groups.

**Table 3:** Univariate and multivariate logistic regression analysis for detecting non-dipper status.

	**Univariate OR (95% CI)**	***P*** ** value**	**Multivariate OR (95% CI)**	***P*** ^*^ **value**
Clinical SBP	1.029(1.002-1.057)	0.033	1.043(1.007-1.080)	0.018
TyG index	9.029(4.196-19.429)	<0.001	9.757(3.929-24.226)	<0.001
HOMA-IR	1.796(1.322-2.440)	<0.001	2.286(1.513-3.455)	<0.001
RDW	1.811(1.293-2.537)	<0.001	1.864(1.203-2.887)	0.005
LDL cholesterol	1.015(1.005-1.024)	0.002	1.018(1.005-1.031)	0.006
24-hour mean BP	1.116(1.071-1.164)	<0.001	1.143(1.080-1.209)	<0.001

Abbreviations: BP, blood pressure; CI, confidence interval; HOMA-IR, homeostasis model assessment of insulin resistance; LDL, low density cholesterol; OR, odds ratio; RDW, red cell distribution width; SBP, systolic blood pressure; TyG, triglyceride-glucose index. * Logistic regression analysis was used.

## Discussion

 The results of the current study showed that the TyG index was higher in non-DP patients than in DP patients with newly diagnosed drug-naive hypertensive patients. Clinical SBP, RDW, LDL cholesterol, 24-hour mean BP, HOMA-IR, and TyG index were independent predictors of non-DP among these patients. The TyG index was negatively associated with the decrease of both SBP and DBP from daytime to nighttime. Furthermore, the AUC value of the TyG index was superior to fasting triglyceride, fasting glucose, and HOMA-IR in detecting non-DP status.

 Hypertension is a well-recognized risk factor for cardiovascular disease, stroke, and target organ damage.^13^ Especially, this risk increases in correlation with an increase in BP.^14^ Compared to clinic BP measurements, much information could be obtained by 24-hour ABPM, including mean BP level, diurnal variation, and BP variability. Within a 24-hour circadian cycle, 24-hour ABPM can discriminate hypertensive patients based on DP and non-DP.^15^ Non-DP BP is more closely associated with cardiovascular events than DP BP.^16,17^ Non-DP BP has been shown to be associated with several clinical conditions such as autonomic dysfunction, chronic kidney disease, connective tissue disease, malignancy, hypothyroidism, and chronic inflammation.^18^ Non-DP was also found to be more common among patients with older age, high salt intake, high stress, poor sleep quality, obesity, and metabolic syndrome.^19^

 IR, which is considered as the major pathologic underlying mechanism of metabolic syndrome, may have a key role in the link between hypertension and metabolic diseases.^20^ It is noted that the presence of IR is associated with an increased risk of hypertension.^21^ The pathologic link between the development of hypertension and IR might be explained by impaired endothelium-dependent vasodilatation, enhanced response to endogenous vasoconstrictors, sympathetic nervous system activation, increased sodium reabsorption in kidneys, and anti-diuretic effect of insulin.^22^ Diabetic patients are at a higher risk for developing non-DP BP.^23^ Tartan et al reported that patients with a higher metabolic syndrome score had more frequent non-DP. Similarly, Mea et al showed that patients with non-DP BP tend to have higher IR, which was assessed by HOMA-IR and adiponectin levels, than patients with DP BP.^24^ The detection of IR plays a pivotal role in the prevention of hypertension and as well as in considering therapy modalities for hypertension.^25^ HOMA-IR, insulin level, and insulin-to-glucose ratio were used to evaluate IR, all of them were found positively correlated with the risk of hypertension.^26^ We showed in this study that non-dipper patients had higher HOMA-IR, which was detected as an independent predictor of patients with a non-DP in the current study.

 TyG index has been widely investigated as a marker of IR in the literature and its association with cardiovascular diseases and adverse events has been evaluated in previous studies. Jian et al concluded that the TyG index was found to be significantly related to the risk of hypertension.^8^ In a meta-analysis consisting of eight observational studies, Wang et al reported that patients with a high TyG index had a 1.53-fold increased risk of developing hypertension.^27^ TyG index was reported to be associated with subclinical arterial stiffness.^28^ Furthermore, the TyG index was also superior to HOMA-IR in predicting the incidence of carotid atherosclerosis. ^29^ Similarly, the TyG index was found superior to HOMA-IR in detecting non-DP in our study. Sanchez-Inigo et al reported that the TyG index was related to the development risk of cardiovascular events.^30^ In accordance with this study, Wang et al reported that the TyG index was independently correlated with adverse events after acute coronary syndrome in diabetic patients.^31^ In this study, we identified a statistically significant difference in TyG index between patients with and without DP BP. The TyG index was also independently linked with non-DP in hypertensive patients. According to our results, the TyG index appears to be an effective marker for detecting non-DP BP in hypertensive patients.

 RDW and LDL-cholesterol were also found as independent predictors of non-DP in our study. RDW, which is the heterogeneity in the measure of erythrocytes, reflects enhanced inflammation and has been suggested as a prognostic indicator in cardiovascular diseases.^32^ The relationship between inflammation and non-DP was presented in a previous report.^33^ Ozcan et al reported that RDW was an independent predictor of non-DP, which was similar to our results.^34^ There were contradictory reports on the association of dyslipidemia with the non-DP. Sunbul et al reported that hyperlipidemia was an independent predictor of non-DP.^35^ In contrast, Chotruangnapa et al could find an independent relation between dyslipidemia and non-DP.^36^ LDL-cholesterol was independently correlated with non-DP in our study, which might suggest the link between metabolic syndrome and non-DP.

 Our study results are valuable for daily clinical practice. Since detecting hypertensive patients who are at high risk is crucial for initiating preventive treatment besides anti-hypertensive treatment, an easily calculable TyG index could provide to identify hypertensive patient’s cardiovascular risk as a better marker of IR than HOMA-IR. Non-DP patients with higher TyG index levels might be more prone to cardiovascular adverse events than those with lower TyG index.

 The major limitations of our study were retrospective design and a single-center study. Due to the cross-sectional study design, there was a lack of inference of causality of results. The other limitation was that fasting blood glucose, fasting triglyceride, and TyG index were measured once at baseline, and we could not get information about the effect of changes in these variables by the follow-up on 24-hour ABPM measurements. The results of the study may have been misestimated because only patients with high clinical BP who underwent 24-hour ABPM were taken and those without 24-hour ABPM were excluded. Finally, this study was conducted in one regional area. Thus, our findings might not be applicable to other areas.

## Conclusion

 In this investigation, we found that the non-DP patients had a higher TyG index, and it was an independent predictor of non-DP among these patients. Additionally, the AUC value of the TyG index was superior to fasting glucose, fasting triglyceride, and HOMA-IR in the discrimination of non-DP BP.

## Funding

 This research did not receive any specific grant from funding agencies in the public, commercial, or not-for-profit sectors.

## Ethical approval

 This research was approved by the ethics committee of Van Training and Research Hospital (approval number: 2021/18).

## Competing interest

 The authors have no conflict of interest to declare.

## References

[1] Williams B, Mancia G, Spiering W, Agabiti Rosei E, Azizi M, Burnier M (2018). 2018 ESC/ESH Guidelines for the management of arterial hypertension. Eur Heart J.

[2] Parati G, Stergiou G, O’Brien E, Asmar R, Beilin L, Bilo G (2014). European Society of Hypertension practice guidelines for ambulatory blood pressure monitoring. J Hypertens.

[3] Ormazabal V, Nair S, Elfeky O, Aguayo C, Salomon C, Zuñiga FA (2018). Association between insulin resistance and the development of cardiovascular disease. Cardiovasc Diabetol.

[4] Marcovecchio ML, Patricelli L, Zito M, Capanna R, Ciampani M, Chiarelli F (2006). Ambulatory blood pressure monitoring in obese children: role of insulin resistance. J Hypertens.

[5] Navarro-González D, Sánchez-Íñigo L, Pastrana-Delgado J, Fernández-Montero A, Martinez JA (2016). Triglyceride-glucose index (TyG index) in comparison with fasting plasma glucose improved diabetes prediction in patients with normal fasting glucose: the Vascular-Metabolic CUN cohort. Prev Med.

[6] Vasques AC, Novaes FS, de Oliveira Mda S, Souza JR, Yamanaka A, Pareja JC (2011). TyG index performs better than HOMA in a Brazilian population: a hyperglycemic clamp validated study. Diabetes Res Clin Pract.

[7] da Silva A, Caldas APS, Hermsdorff HHM, Bersch-Ferreira ÂC, Torreglosa CR, Weber B (2019). Triglyceride-glucose index is associated with symptomatic coronary artery disease in patients in secondary care. Cardiovasc Diabetol.

[8] Jian S, Su-Mei N, Xue C, Jie Z, Xue-Sen W (2017). Association and interaction between triglyceride-glucose index and obesity on risk of hypertension in middle-aged and elderly adults. Clin Exp Hypertens.

[9] Lambrinoudaki I, Kazani MV, Armeni E, Georgiopoulos G, Tampakis K, Rizos D (2018). The TyG index as a marker of subclinical atherosclerosis and arterial stiffness in lean and overweight postmenopausal women. Heart Lung Circ.

[10] Chissini RBC, Kuschnir MC, de Oliveira CL, Giannini DT, Santos B (2020). Cutoff values for HOMA-IR associated with metabolic syndrome in the Study of Cardiovascular Risk in Adolescents (ERICA Study). Nutrition.

[11] Verdecchia P, Schillaci G, Porcellati C (1991). Dippers versus non-dippers. J Hypertens Suppl.

[12] Zheng R, Mao Y (2017). Triglyceride and glucose (TyG) index as a predictor of incident hypertension: a 9-year longitudinal population-based study. Lipids Health Dis.

[13] Kjeldsen SE (2018). Hypertension and cardiovascular risk: general aspects. Pharmacol Res.

[14] Kannel WB (2000). Elevated systolic blood pressure as a cardiovascular risk factor. Am J Cardiol.

[15] Dai S, Huang B, Zou Y, Liu Y (2019). Associations of dipping and non-dipping hypertension with cardiovascular diseases in patients with dyslipidemia. Arch Med Sci.

[16] Fagard RH, Celis H, Thijs L, Staessen JA, Clement DL, De Buyzere ML (2008). Daytime and nighttime blood pressure as predictors of death and cause-specific cardiovascular events in hypertension. Hypertension.

[17] Wei FF, Li Y, Zhang L, Xu TY, Ding FH, Staessen JA (2014). Association of target organ damage with 24-hour systolic and diastolic blood pressure levels and hypertension subtypes in untreated Chinese. Hypertension.

[18] Verdecchia P, Schillaci G, Reboldi G, Franklin SS, Porcellati C (2001). Different prognostic impact of 24-hour mean blood pressure and pulse pressure on stroke and coronary artery disease in essential hypertension. Circulation.

[19] Dubielski Z, Zamojski M, Wiechecki B, Możeńska O, Petelczyc M, Kosior DA (2016). The current state of knowledge about the dipping and non-dipping hypertension. Arter Hypertens.

[20] Zhou MS, Wang A, Yu H (2014). Link between insulin resistance and hypertension: what is the evidence from evolutionary biology?. Diabetol Metab Syndr.

[21] Bonora E, Kiechl S, Willeit J, Oberhollenzer F, Egger G, Targher G (1998). Prevalence of insulin resistance in metabolic disorders: the Bruneck Study. Diabetes.

[22] Kotchen TA (2010). Obesity-related hypertension: epidemiology, pathophysiology, and clinical management. Am J Hypertens.

[23] Lurbe E, Redon J, Kesani A, Pascual JM, Tacons J, Alvarez V (2002). Increase in nocturnal blood pressure and progression to microalbuminuria in type 1 diabetes. N Engl J Med.

[24] Della Mea P, Lupia M, Bandolin V, Guzzon S, Sonino N, Vettor R (2005). Adiponectin, insulin resistance, and left ventricular structure in dipper and nondipper essential hypertensive patients. Am J Hypertens.

[25] Tartan Z, Uyarel H, Kasikcioglu H, Alper AT, Ozay B, Bilsel T (2006). Metabolic syndrome as a predictor of non-dipping hypertension. Tohoku J Exp Med.

[26] Arshi B, Tohidi M, Derakhshan A, Asgari S, Azizi F, Hadaegh F (2015). Sex-specific relations between fasting insulin, insulin resistance and incident hypertension: 89 years follow-up in a Middle-Eastern population. J Hum Hypertens.

[27] Wang Y, Yang W, Jiang X (2021). Association between triglyceride-glucose index and hypertension: a meta-analysis. Front Cardiovasc Med.

[28] Won KB, Park GM, Lee SE, Cho IJ, Kim HC, Lee BK (2018). Relationship of insulin resistance estimated by triglyceride glucose index to arterial stiffness. Lipids Health Dis.

[29] Irace C, Carallo C, Scavelli FB, De Franceschi MS, Esposito T, Tripolino C (2013). Markers of insulin resistance and carotid atherosclerosis A comparison of the homeostasis model assessment and triglyceride glucose index. Int J Clin Pract.

[30] Sánchez-Íñigo L, Navarro-González D, Fernández-Montero A, Pastrana-Delgado J, Martínez JA (2016). The TyG index may predict the development of cardiovascular events. Eur J Clin Invest.

[31] Wang L, Cong HL, Zhang JX, Hu YC, Wei A, Zhang YY (2020). Triglyceride-glucose index predicts adverse cardiovascular events in patients with diabetes and acute coronary syndrome. Cardiovasc Diabetol.

[32] Fava C, Cattazzo F, Hu ZD, Lippi G, Montagnana M (2019). The role of red blood cell distribution width (RDW) in cardiovascular risk assessment: useful or hype?. Ann Transl Med.

[33] Kaya MG, Yarlioglues M, Gunebakmaz O, Gunturk E, Inanc T, Dogan A (2010). Platelet activation and inflammatory response in patients with non-dipper hypertension. Atherosclerosis.

[34] Özcan F, Turak O, Durak A, İşleyen A, Uçar F, Giniş Z (2013). Red cell distribution width and inflammation in patients with non-dipper hypertension. Blood Press.

[35] Sunbul M, Gerin F, Durmus E, Kivrak T, Sari I, Tigen K (2014). Neutrophil to lymphocyte and platelet to lymphocyte ratio in patients with dipper versus non-dipper hypertension. Clin Exp Hypertens.

[36] Chotruangnapa C, Tansakun T, Roubsanthisuk W (2021). Clinical risk factors and predictive score for the non-dipper profile in hypertensive patients: a case-control study. Clin Hypertens.

